# Postpartum Depression Epidemiology, Risk Factors, Diagnosis, and Management: An Appraisal of the Current Knowledge and Future Perspectives

**DOI:** 10.3390/jcm14072418

**Published:** 2025-04-01

**Authors:** Zaituna Khamidullina, Aizada Marat, Svetlana Muratbekova, Nagima M. Mustapayeva, Gulnar N. Chingayeva, Abay M. Shepetov, Syrdankyz S. Ibatova, Milan Terzic, Gulzhanat Aimagambetova

**Affiliations:** 1 Department of Obstetrics and Gynecology #1, NJSC “Astana Medical University”, Astana 010000, Kazakhstan; zaituna59@gmail.com (Z.K.); marat.a@amu.kz (A.M.); 2 Higher School of Medicine, NJSC Sh. Ualikhanov Kokshetau University, Kokshetau 020000, Kazakhstan; muratbekova.s@mail.ru; 3 Department of Nephrology, Asfendiyarov Kazakh National Medical University, Almaty 050000, Kazakhstan; mustapayeva.n@kaznmu.kz (N.M.M.); chingayeva.g@kaznmu.kz (G.N.C.); shepetov.a@kaznmu.kz (A.M.S.); 4 JSC “National Neurosurgery Center”, Astana 010000, Kazakhstan; goha_0870@mail.ru; 5 Department of Surgery, School of Medicine, Nazarbayev University, Astana 010000, Kazakhstan; milan.terzic@nu.edu.kz; 6 Clinical Academic Department of Women’s Health, CF University Medical Center, Astana 010000, Kazakhstan

**Keywords:** postpartum depression, perinatal depression, maternal mental health, Edinburgh Postnatal Depression Scale, risk factors, pharmacological treatment, cognitive behavioral therapy, interpersonal therapy

## Abstract

Postpartum depression (PPD) is a severe mental health condition that affects women following childbirth and is marked by persistent sadness, anxiety, fatigue, and difficulty functioning. Unlike the temporary “baby blues”, PPD is more severe and long-lasting, potentially leading to negative consequences for mother and child. Globally, PPD impacts approximately 10–20% of postpartum women, with prevalence influenced by genetic, hormonal, psychological, and socio-environmental factors. Early detection is crucial, with screening tools such as the Edinburgh Postnatal Depression Scale (EPDS) commonly used in clinical practice. Treatment options include pharmacological interventions such as selective serotonin reuptake inhibitors (SSRIs), psychological therapies like cognitive behavioral therapy (CBT) and interpersonal therapy (IPT), and lifestyle modifications. Despite the growing awareness of PPD, stigma remains a significant barrier to treatment, discouraging many women from seeking help. In low-income countries, where mental health care is often underfunded, accessing professionals trained in perinatal mental health presents an even greater challenge. This gap underscores the urgent need for a collaborative, multidisciplinary approach involving obstetricians, psychiatrists, pediatricians, and midwives to ensure comprehensive support and care for affected individuals.

## 1. Introduction

Maternal mental health problems are a significant complication of pregnancy and the postpartum period and are frequently encountered by healthcare professionals in their clinical practice [1,2,3,4]. The postpartum period is crucial for women and their offspring’s current and future well-being and is an important time in a woman’s life. It features major hormonal changes affecting emotional status, with the potential to predispose women to postpartum mental health disorders.

Postpartum depression (PPD) is a serious psychiatric condition that occurs within one year after childbirth [5]. This term includes both prenatal and postpartum depression. According to the World Health Organization (WHO), more than 80% of women may face a combination of emotional difficulties during pregnancy and after delivery [6,7]. PPD is one of the most common incapacitating complications of childbearing that negatively impacts the mother. The condition is often neglected and underdiagnosed; thus, suffering mothers do not receive appropriate treatment [4]. As a result, suicide following non-treated PPD accounts for approximately 20% of postpartum deaths [8]. Women with severe depression may exhibit unhealthy general behavior and eating habits that might affect their newborns [9]. Moreover, maternal mental health issues also have an impact on offspring, such as adverse effects on a child’s cognitive, behavioral, and emotional development [10,11,12]. Recent scientific evidence into the pathophysiology of PPD and its management may offer potentially effective therapeutic options [4].

Understanding stress and depression disorders related to giving birth could provide a valuable opportunity to properly manage the existing perinatal mental health issues and prevent PPD in the future. This scoping review aims to summarize and disseminate the current research findings on postpartum depression’s prevalence, risk factors, diagnosis, and management.

## 2. Materials and Methods

A literature review was conducted using the PubMed, Ebsco, Google Scholar, and Cochrane databases in March 2025. The search was performed using the following search terms: “postpartum period”, “depression”, “postpartum depression”, “delivery”, “pregnancy”, “mental health”, “maternal mental health”, “perinatal”, “postnatal”, “well-being”, “risk factors”, “epidemiology”, “prevalence”, “incidence”, “diagnosis”, “management”.

Medical subject heading (MeSH) terms were used whenever available: “postpartum period” (MeSH Unique ID: D049590), “postpartum depression” (MeSH Unique ID: D019052), “maternal behavior” (MeSH Unique ID: D008425), “mental health” (MeSH Unique ID: D008603). Boolean operators (AND, OR) were used to combine keywords. If search terms were incomplete, an additional search was performed using modified search terms. Supplementary Table S1 shows the keywords used and search threads generated.

Overall, 509 articles were identified with the initial screening. The titles and abstracts of articles were collected using the search strategy and were reviewed by the authors to identify studies that could potentially align with the study’s objectives. Duplicate and irrelevant articles that did not meet the specified search criteria were excluded. The full texts of potentially eligible studies were then obtained and assessed for relevance. Finally, 117 articles were included in this review.

## 3. Definition and Epidemiology of Postpartum Depression

Postpartum depression refers is a transient psychological condition that occurs within 12 months after childbirth [1,2,3,4,13]. The pregnancy and postpartum periods are thought to be specifically vulnerable periods for the manifestation of maternal mental health issues, including depression [1,2,14]. As one of the most common mental disorders occurring during the perinatal period, PPD impacts the functional ability of a woman in many domains of life and causes complex negative effects on children as well as parental and marital relationships [15,16,17,18]. Thus, PPD is a severe psychiatric disorder that is underdiagnosed and underestimated in many cases, leading to serious complications including suicide.

Reports on the worldwide prevalence of PPD provide a controversial and wide range of data. Some studies report the estimated overall prevalence of PPD at approximately 10–17% (Table 1) [18,19,20,21,22], while other studies provide data indicating a very high prevalence of up to 60% [21,23]. The prevalence of PPD is reported to be higher in low- and middle-income countries (LMICs) than in high-income countries [2,24,25,26]. In developing countries, around 20% of women suffer from PPD. Some other epidemiological reports show that the PPD prevalence in developed countries ranges from 7% to 40%, and in Asian countries ranges from 3.5% to 63.3% [21,23]. The results of a systematic review that included studies conducted in developed countries suggest that more than 19% of new mothers might suffer from PPD in the first 12 weeks of the postpartum period, with up to 7% developing major depression [19]. A meta-analysis focusing on LMICs shows that the prevalence of non-psychotic common postpartum mental disorders is close to 20% [27].

The prevalence of PPD is also different depending on ethnic and cultural differences [30]. A study on the prevalence of PPD among European women reported the prevalence of PPD as up to 55% [28]. In China, the incidence rate of PPD was reported to be between 1% and 52.1%, with an average of 14.7% [21]. A study from Japan shows that more than 10% of mothers in Japan experienced depressive symptoms identified through the routine utilization of the Edinburgh Postnatal Depression Scale (EPDS) [31]. Another Japanese study reports that PPD occurs in 10–15% of postpartum women in the country [32]. A survey conducted in Australia among immigrant women from Afghanistan showed that the prevalence of PPD was as high as 30% [29,33]. The PPD prevalence was found to be high among Chinese immigrant women as well [29].

Notably, most women with PPD recover within a few months; however, approximately 30% of women later develop major depression and suffer beyond the first year after childbirth [29,34]. Moreover, the risk of PPD recurrence in the postpartum period of subsequent pregnancies or non-postpartum depression episodes is approximately 40% [29].

## 4. Pathophysiology and Risk Factors of Postpartum Depression

**Figure 1:**
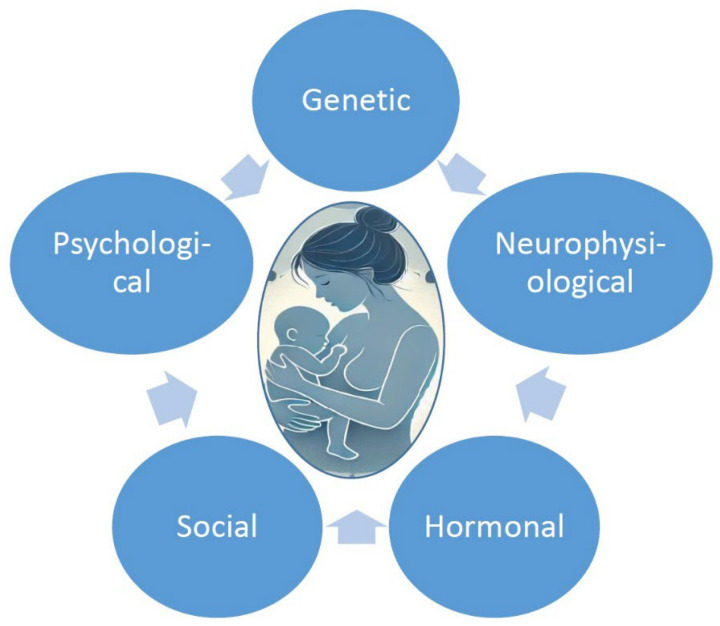
Risk factors of postpartum depression.

There are multiple factors and mechanisms implicated in the pathophysiology of PPD, including genetic and biochemical factors and neuroinflammatory changes [35,36]. Various non-specific factors, such as previous negative life experiences, a history of psychological issues (stress, depression, and anxiety disorders), social and cultural specifics, marital and/or financial challenges, psychological features, and stress-coping skills, have an impact on the risk of PPD development (Figure 1) [25]. Multiple specific risk factors for PPD have been identified including prenatal maternal depression and anxiety, impaired infant–mother contact and bonding, and lack of spouse/family support [1,2]. Cultural differences involving various beliefs or actions related to cultural/ethnic/personal understanding of PPD, a lack of understanding between spouses, or an adverse reaction to a postpartum mother’s depressive symptoms from their partner’s side may be important risk factors for depression and contribute to PPD’s appearance and development.

The possibility that previous negative life events are risk factors for PPD is currently of particular interest [35,36,37]. Mothers who experienced multiple adverse life events such as childhood or adulthood sexual abuse were found to be at increased risk of PPD. This group of patients is three times more likely to have PPD in comparison with those who did not encounter any adverse life events [37]. Chronic stress during pregnancy also predisposes mothers to depression and anxiety in the postpartum period and is associated with deficits in the maternal care of newborns and impaired mother–child bonding [35,36,38,39,40,41].

Studies of twins and families report the influence of genetic factors on the development of PPD [42,43]. Genome-wide association studies have revealed candidate genes and potential pathways involved in PPD [44]. The estrogen receptor alpha gene plays a role in mediating hormonal changes during pregnancy and the postpartum period. It makes the estrogen receptor alpha gene an interesting candidate for genetic association studies in PPD [44,45]. Furthermore, promising evidence has linked mutations in the serotonin transporter with PPD [46,47,48]. Studies suggest that a lower expression of the serotonin transporter gene makes women more predisposed to PPD symptoms after childbirth [49].

Monoamine oxidase A (MAOA), an enzyme implicated in the oxidative de-amination of amines, such as dopamine, norepinephrine, and serotonin, was also found to play a role in PPD development (Table 2) [50,51,52]. Polymorphisms in the MAOA gene have been found to have an association with PPD, and variants of MAOA have been positively correlated with the severity of PPD scores [50,51,52].

Certain neuroendocrine mechanisms play a role in the development of PPD. The peripartum period is a time of sudden and dramatic changes in hormone production. This period is also a time of specific susceptibility to the development of psychological/mood disorders. It is believed that these two processes are associated with fluctuations in reproductive hormones that may play a role in the underlying neurobiology of postpartum mood disorders. The concept has led to the “ovarian-steroid-withdrawal hypothesis” [62,63]. Further, neuroendocrine abnormalities, such as elevated levels of stress hormones during the peripartum period, have also been implicated in the underlying neurobiology of postpartum mood disorders.

There are a number of findings that imply the hypothalamic-pituitary-adrenal (HPA) axis’s involvement in postpartum depression [54]. This is based on the fact that stress is a prominent risk factor for postpartum depression, and neuroendocrine disruptions are one of the most consistent findings in major depressive disorder [53,54]. Consistent with the role of HPA axis dysfunction in PPD, there is evidence of altered levels of cortisol, ACTH, and CRH in patients suffering from postpartum depression [44]. Elevated levels of corticotrophin-releasing hormone (CRH) have even been suggested to be a diagnostic criterion for postpartum depression [54].

Estrogen levels rise dramatically before parturition, reaching levels over 1000 times their baseline values, and then precipitously drop after delivery. Changes in absolute estradiol levels have not been consistently reported in patients with PPD [45]. However, it has been suggested that women with PPD may exhibit increased sensitivity to estrogen signaling based on changes in estrogen-sensitive transcript expression [45]. Furthermore, estrogen signaling is known to impact other pathways involved in mood, such as the HPA axis. Several studies suggest that estrogen treatment reduces the risk of developing PPD [55,56,64]. In contrast to the antidepressant effects of estrogen, progesterone treatment has been shown to increase the risk and worsen depression in postpartum women [55,57,58].

Oxytocin also plays an important role in postpartum depression as it is a well-known factor for the regulation of emotions, social interaction, stress, and mother–infant relationships, including delivery, lactation, and attachment [59,60]. However, the oxytocin–breastfeeding–depression relationship is not fully clarified yet, with some conflicting results in recent studies [58]. Some studies have shown oxytocin levels during breastfeeding to be inversely correlated with depression symptoms [65]. However, in one study, intranasal oxytocin treatments failed to improve maternal sensitivity measures [66]. More recently, researchers have reported that maternal venous oxytocin levels during breastfeeding, measured 2 and 6 months into the postpartum period, showed no significant variation based on depression symptom status [58,67].

Evidence of a relationship between breastfeeding and PPD is well established [58]. Prolactin has a well-known role in lactation and maternal behaviors. Researchers report that hyperprolactinemia may induce PPD, although the exact mechanism of this process is not well understood [68,69]. Other studies have reported lower PPD risk associated with a longer reported breastfeeding duration [58]. Both meta-analyses suggest that the relationship between breastfeeding and PPD is varied by the type of breastfeeding (exclusive vs. partial breastfeeding) [58].

It should also be noted that thyroid hormones have also been implicated in PPD [35,61]. Thyroid hormones have been suggested as a marker of PPD due to the proven role of thyroid dysfunction in major depression [64]. Moreover, it is well known that thyroid function flaws are associated with pregnancy and, thus, could potentially contribute to PPD.

The potential mechanisms discussed above do not function separately but are extremely interrelated. It is likely that numerous factors may collectively contribute to PPD development. One of the predominant risk factors for the development of postpartum depression is stress and previous adverse life events, which are linked to neuroendocrine dysfunction associated with PPD. Understanding the underlying pathophysiology of PPD may help in understanding the condition and finding appropriate management options.

## 5. Symptoms and Diagnosis of Postpartum Depression

Timely diagnosis and management of PPD are critical for ensuring maternal health and supporting the developmental well-being of the newborn/child. The diagnostic criteria for PPD include a “combination of depressed mood, loss of interest, anhedonia, sleep and appetite disturbance, impaired concentration, psychomotor disturbance, fatigue, feelings of guilt or worthlessness, and suicidal thoughts, which are present during the same two-week period and are a change from previous functioning” [4]. The transition to motherhood can result in some mothers feeling insecure and showing symptoms of stress and anxiety. PPD has several temporary symptoms such as brief crying spells, tearfulness, irritability or emotional lability, sorrow/weeping, unstable mood, insomnia, anxiety, loss of appetite, and poor concentration [3,4,41]. These symptoms must cause clinically significant distress and have an impact on functioning that is “not attributable to a substance or to another medical condition” [4].

Overall, PPD reduces maternal mental health and quality of life. Women experiencing PPD appear to be anxious and grumpy and struggle with routine household tasks. They have negative feelings about themselves and their children, appear tearful, and may experience marital problems [3,4]. In addition, women with PPD could have physical symptoms, such as sleep and appetite disturbances, and display obsessional behavior [3,4,70]. The quality of life for such women and their families is severely compromised, which can result in marital breakdown [71,72,73]. In the most severe cases, women report fear of hurting themselves or their newborns [74,75,76]. According to studies, 5–14% of perinatal and postnatal women have thoughts of self-harm, and suicides account for up to 20% of postpartum deaths [8,76,77]. Thus, proper screening for PPD may help to improve not only mental but physical well-being as well.

The identification of these symptoms and confirmation of PPD is a sensitive clinical inquiry for obstetricians and primary care providers during the postpartum period [4]. A screening instrument that identifies the risk of depression during pregnancy and the postpartum period would help to prevent aggravation of PPD even when it occurs. Many mental health screening tools have been validated to measure perinatal mental health issues [78]. Currently, 12 available instruments have been designed to assess the risk factors of PPD [70]. These instruments are based on measuring different factors combined with prenatal, perinatal, and postnatal risk factors for PPD.

Since 1978, when the first checklist to identify the risk factors for PPD in pregnancy became available [79], several similar instruments have been developed. The Postpartum Depression Predictors Inventory-Revised (PDPI-R) [80] is the antenatal screening scale whose design has been based on the findings of meta-analyses [81]. This was the first instrument to assess risk factors that occur during both pregnancy and the postpartum period. The PDPI-R was originally designed to be administered via an interview conducted by a clinician.

The Beck Depression Inventory (BDI) is another tool used to measure PPD [82,83]. However, the accuracy of the BDI-II in the accurate detection of PPD is controversial [84]; therefore, the BDI-II instrument should be used with caution during PPD diagnosis [83]. Researchers and clinicians who screen for PPD should “pay particular attention to cognitive/affective symptoms, as they appear more robust to normative physical and emotional changes that occur in the postpartum” [83]. For BDI, a score of 17 or more suggests the appearance of depression symptoms. Different levels of depression, from borderline to extreme depression, could be categorized using BDI. However, specifically for BDI-II, researchers suggest a cut-off score of >12 as a criterion to indicate “mild depression in the postpartum period” [83]. At present, the Edinburgh Postnatal Depression Scale is considered the gold standard in screening for PPD [25,85,86,87,88]. The validation and analysis of the EPDS’s items have been carried out in a large variety of studies/samples and in different countries and cultures [78,86,89,90,91,92,93]. Several studies have obtained a combination of three factors: depressive or non-specific depressive symptoms (items 7, 8, 9, and 10), anhedonia factor (items 1 and 2), and anxiety symptoms (items 3, 4, and 5) [94]. These results imply that obtaining an anxiety dimension using items from a scale designed to assess depressive symptoms poses construct validity problems for the EPDS. A score of more than 10 suggests minor or major depression; thus, further evaluation is recommended. The use of the EPDS as a “gold standard” of postpartum depression may minimize the importance of postpartum anxiety symptoms. Compared to other scales used to measure PPD, the EPDS takes only few minutes to complete, is freely accessible, and has been validated for use throughout the perinatal period (during pregnancy and in the postpartum period). The English version of the EPDS has good internal consistency and reliability [85]. Moreover, it has been translated into 50 languages and passed through multiple cross-cultural validation studies.

The Depression, Anxiety, and Stress Scale (DASS) is a 21-item self-reported instrument for measuring the negative emotional states of depression, anxiety, and stress [95]. Some studies use DASS-21 to measure PPD levels. These studies report that DASS-21 shows good internal reliability for measuring PPD [78,96]. The DASS-21 questionnaire demonstrates a “satisfactory ability to discriminate cases from non-cases” [78]. These findings suggest that the DASS-21 instrument could be used as an alternative tool when screening PPD. It has fewer questions, which is considered to be an advantage of the instrument [78]. However, it should be noted that the DASS-21 scale is not diagnostic, but measures the severity of symptoms. Compared to EPDS, DASS-21 should be used as part of a comprehensive PPD assessment.

There are many more instruments that have been validated and utilized for PPD diagnosis; however, when choosing between one and another, their reliability and cultural validation results should be taken into consideration. Currently, the majority of studies/researchers and clinicians prefer using EPDS [25].

## 6. Association of Maternal Postpartum Depression with Children’s Development

**Figure 2:**
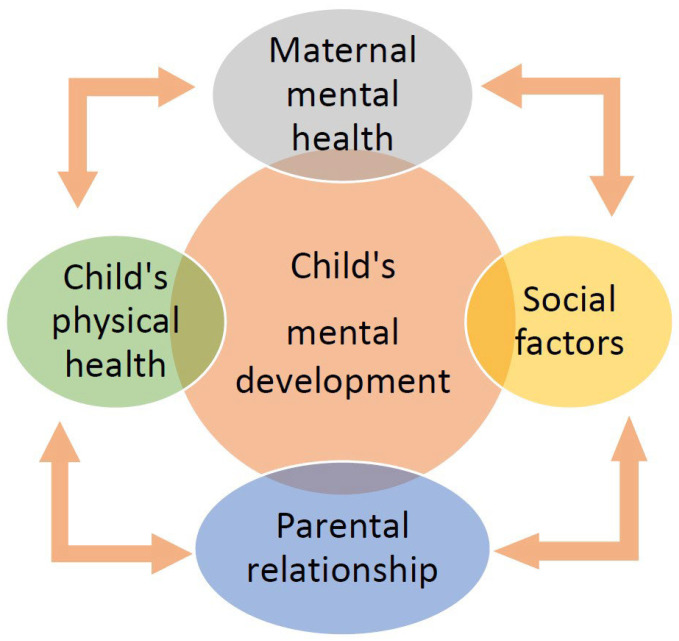
Impact of postpartum depression on children’s development.

PPD, being a common mental health disorder, could also be associated with long-lasting effects for the children of affected women. The developmental curves of these children, including cognitive development, have been noted to be affected by their mother’s mental health and well-being (Figure 2) [40,41]. Many research studies have described the impact of PPD on the health and well-being of the mother and its effects on the health outcomes of the infant [40,97]. PPD has a negative influence on child health and development because it interferes with the mother’s ability to care for a baby and handle other daily tasks, and the mother–child relationship often worsens because of PPD [40,98]. Maternal depression during the 4–6 month postpartum period has been found to be significantly related to adequate newborn development [25,40]. Mothers with a diagnosis of PPD have been found to have decreased sensitivity to their infant’s cues, such as crying; thus, newborns’ well-being and needs could be neglected [25,40].

Studies focused on the later developmental stages of children from mothers with postpartum mental disorders have shown a direct relationship between maternal depression and negative cognitive outcomes in children at preschool and school ages [40,99,100,101,102]. Studies report a direct relationship between the amount of “cognitive stimulation provided by the mothers towards their children at 5 and 8 years of age” and their performance on cognitive tests [40,103]. Although PPD may have a lower impact on children’s intellectual development in adolescent ages in comparison to other ages, maternal interest and support were found to have a strong connection to the child’s achievements.

A decrease in maternal sensitivity towards infant cues has been associated with a decrease in cognitive stimulation which, therefore, could impact intellectual development in the child [40,41]. Moreover, severe depression is also reported to be associated with child abuse [25,41]. Therefore, to protect offspring, all women during pregnancy and the postpartum period should be quickly and appropriately screened for PPD [40]. Early and accurate identification and intervention must occur to prevent long-term consequences for childbearing families and to prevent PPD from being converted into a significant mental health disorder.

## 7. Management of Postpartum Depression

**Figure 3:**
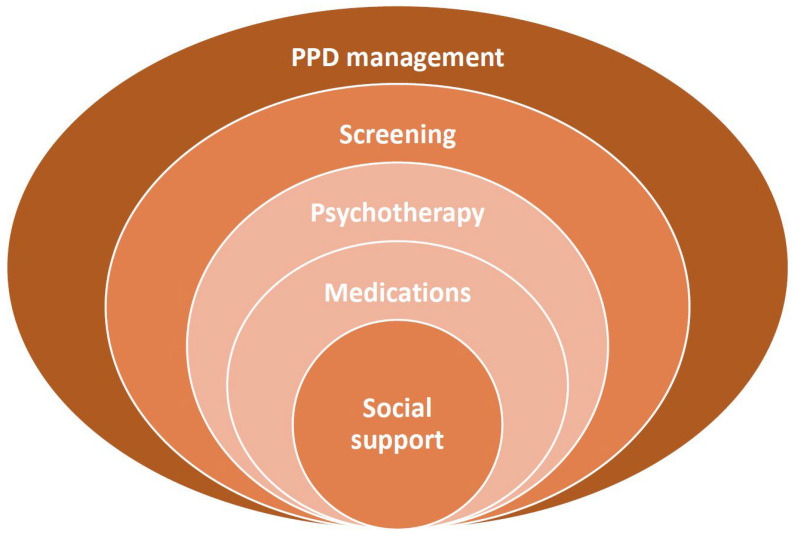
Multilayer management of postpartum depression.

The management of PPD typically involves a combination of different treatment methods: psychological, pharmacological, and lifestyle interventions, as well as family and social support (Figure 3) [7,104,105,106,107,108]. The approach depends on the expressiveness of symptoms, the severity of the condition, and individual patient needs [41]. 

Psychotherapy is often the first-line treatment, especially for mild to moderate PPD. Cognitive behavioral therapy (CBT) is a structured, time-limited psychotherapy that focuses on identifying and modifying negative mind patterns and behaviors. It is effective in reducing depressive symptoms in postpartum women [104,105,107,109]. A study by Li et al. (2022) found CBT to be effective for the management of maternal anxiety, stress, and PPD [105]. A more recent meta-analysis also reports that CBT-based facilitations for depression during the perinatal period appear to be effective [110].

Another option is interpersonal therapy (IPT), which aims to address interpersonal issues and role transitions that may contribute to depression. Studies have demonstrated its efficacy in treating postpartum depression by improving interpersonal relationships and social functioning [107,109,111]. A study of HIV-positive pregnant women with mental health issues found that IPT was effective in the management of PPD [106]. However, limited studies on this method of management are available for review.

The use of peer support groups as a management option for PPD provides a platform for new mothers to share experiences and coping strategies, reducing feelings of isolation and promoting recovery. Research indicates that such groups can be beneficial in managing postpartum depression [107,112]. The limited studies on peer support therapy indicate that this management option provides a potential mechanism for improving mental health outcomes [112]. The method was found to be effective as a preventative strategy for PPD [113]. These approaches are effective mostly for mild and moderate cases of PPD.

The majority of cases of moderate or severe PPD require pharmacotherapy or a combination of psychotherapy and pharmacotherapy. Pharmacological treatments include selective serotonin reuptake inhibitors (SSRIs) and other neuroactive steroid drugs such as gamma-aminobutyric acid (GABA) modulators [107,108,109,114].

SSRIs, such as sertraline and fluoxetine, are commonly prescribed for moderate to severe postpartum depression [107,108]. Most SSRIs (like fluoxetine, sertraline, and escitalopram) have minimal adverse effects on milk production and infant development when used during breastfeeding; thus, they are generally considered safe for breastfeeding mothers, with minimal infant exposure through breast milk.

In August 2023, the United States (USA) Food and Drug Administration (FDA) approved zuranolone as the first oral medication specifically for PPD. Zuranolone acts primarily as a positive allosteric modulator of GABA receptors. Clinical trials have shown that zuranolone significantly reduces depressive symptoms in postpartum women [108,115]. A recent study also reported that zuranolone was well tolerated by patients and demonstrated significant improvements in depressive symptoms [115]. This supports the high potential of the medication for the management of maternal mental health issues as a new rapid-acting pharmacotherapy for PPD.

Brexanolone is a derivative of progesterone metabolite allopregnanolone that works by modulating the brain’s stress response through GABA receptors. It is an intravenous medication approved for the treatment of PPD [114,116]. It has been shown to provide rapid relief of depressive symptoms, though it requires administration in a healthcare setting due to potential side effects. The main disadvantage of the medication is the need for hospital admission, as it is given as a 60 h intravenous infusion in a hospital setting.

Although an amount of zuranolone or brexanolone might transfer into breast milk, the exact concentrations of these medications in breast milk have not been extensively studied. Given the short half-life of brexanolone (9–13 h), the amount transferred to the infant would likely be minimal. However, zuranolone has a half-life of 24 h; thus, it should be used with caution [114,115].

Regarding pharmacotherapy, it should be noted that any medication should be prescribed after weighing risks vs. benefits, especially for breastfeeding mothers. Moreover, it may take 2–4 weeks to notice significant improvements with antidepressants. Some medications may require dose adjustments under medical supervision.

Lifestyle modifications may help to improve the condition of patients with PPD. These include regular exercise, adequate sleep, and a healthy diet [7,9]. Social support is another option to help women with PPD. It involves family and partner engagement and community resources [7]. Educational interventions designed to inform male partners about the symptoms and signs of PPD could improve family support and bonding. Active participation from family members and partners in childcare and household responsibilities can alleviate stress and provide emotional support, which is vital for recovery from postpartum depression. Community-based support services are usually based on home visits from healthcare professionals or local parenting groups, which can offer additional assistance and reduce feelings of isolation [7].

Unfortunately, some severe cases do not respond to pharmacotherapy or other methods of management and, thus, require medical interventions such as electroconvulsive therapy (ECT) [107,117]. For severe cases of PPD, especially those with psychotic features or where other treatments have failed, ECT may be considered. It has been shown to be effective in rapidly alleviating depressive symptoms. Compared to its use in the management of non-postpartum depression or psychosis, ECT shows a higher response rate in PPD cases [117].

The utilization of effective preventive strategies may help to reduce the prevalence of PPD and assist in early diagnosis. Screening is one of the most significant parts of the prevention strategy. Routine screening for depression during pregnancy and the postpartum period is recommended to identify at-risk individuals early. Tools like the EPDS are commonly used for this purpose in many countries [87]. Another important part of the preventive measures is education and awareness [7]. This includes teaching and informing pregnant women about PPD symptoms and signs that could facilitate early recognition and prompt treatment. Moreover, family members should also be informed about how to identify the condition and offer support.

A comprehensive approach that includes psychological support, appropriate pharmacological treatment, lifestyle adjustments, and strong social support networks is essential for effectively managing postpartum depression. Early intervention and personalized care plans are crucial for the well-being of both mother and child.

## 8. Conclusions

PPD is a prevalent and debilitating disorder. While several effective treatment options exist, including pharmacological and psychological therapies, psychosocial interventions, and neuromodulation techniques, many remain under-researched. Unfortunately, the available treatments are significantly underutilized in the community. Although PPD is increasingly recognized and discussed, stigma persists, preventing many women from seeking care. In low-income countries, mental health is often not a priority, and women may face barriers to accessing providers with specialized training in perinatal mental health, even when they seek help. Given the complexity of the problem, a collaborative approach involving multiple healthcare providers such as obstetricians, psychiatrists, pediatricians, and nursing/midwifery professionals is essential. Expanding reproductive psychiatry education within psychiatry residency and fellowship programs is crucial to improving awareness and care.

## Figures and Tables

**Table 1:** Global epidemiology of postpartum depression: prevalence, clinical insights, and socioeconomic influences [19,21,23,27,28,29].

Prevalence/Incidence	Population/Region	Socioeconomic/Clinical Context	Clinical Insights	Socioeconomic/Healthcare Factors	Study/Source
10–17%	Global	Mixed (high- and low-income countries)	General prevalence of PPD	Potential underdiagnosis in low-resource settings	[19]
7% to 40%	Developed countries (including Japan and China)	High-income, better mental health support	Variation in PPD prevalence due to healthcare access	Differences in mental health support and social services	[23]
20%	Low- and middle-income countries	Limited mental health resources, high stigma	High prevalence of non-psychotic postpartum mental disorders	Barriers to accessing care and societal stigma	[27]
3.5% to 63.3%	Asian countries (including China)	Diverse cultural and socioeconomic backgrounds	Cultural beliefs and economic factors influencing PPD	Significant healthcare disparities within the region	[23]
55%	European women	High-income, specific regional contexts	Exceptionally high PPD prevalence in specific populations	Possible influence of cultural and regional stressors	[28]
30%	Afghan immigrant women in Australia	Immigrant group, socio-cultural challenges	Increased PPD risk due to resettlement stress	Potential lack of culturally sensitive support systems	[29]
Up to 60%	Various regions	Mixed (extreme cases in vulnerable groups)	Highest reported PPD prevalence	Often seen in underrepresented or high-risk populations	[21]

PPD—postpartum depression.

**Table 2:** Pathophysiological mechanisms and risk factors of postpartum depression [35,37,38,39,40,41,44,45,51,52,53,54,55,56,57,58,59,60,61,62,63].

Factor/Mechanism	Specific Elements	Clinical Impact	Study/Source
Genetic Factors	Estrogen receptor α gene, serotonin transporter gene, MAOA gene polymorphisms	Increased vulnerability to PPD, particularly after childbirth	[44,45,51,52]
Neuroendocrine Factors	HPA axis dysfunction, neuroinflammatory changes, oxidative stress Estrogen and progesterone fluctuations, oxytocin, prolactin, thyroid hormones, cortisol, ACTH, CRH	Dysregulation of stress response, impact on mood, emotion regulation contribute to PPD symptoms	[35,53,54,55,56,57,58,59,60,61]
Neuroinflammatory Changes	Hormonal withdrawal (ovarian-steroid-withdrawal hypothesis)	Association with mood disorders and increased PPD risk	[62,63]
Psychological and Social Factors	Previous negative life events, stress, anxiety, depression history, marital/financial challenges	Increased risk of PPD, impaired mother–child bonding	[37,38,39,40,41]

ACTH—adrenocorticotropic hormone; CRH—corticotropin-releasing hormone; HPA—hypothalamic-pituitary-adrenal; MAOA—monoamine oxidase A; PPD—postpartum depression.

## Data Availability

The data related to the article are available from the corresponding author upon reasonable request.

## Supplementary Materials

The following supporting information can be downloaded at: https://www.mdpi.com/article/10.3390/jcm14072418/s1, Table S1: Search threads.

## References

[1] O’Hara M.W., McCabe J.E. (2013). Postpartum depression current status and future directions. Annu. Rev. Clin. Psychol..

[2] O’Hara M.W., Wisner K.L. (2014). Perinatal mental illness: Definition, description and aetiology. Best Pract. Res. Clin. Obstet. Gynaecol..

[3] Tosto V., Ceccobelli M., Lucarini E., Tortorella A., Gerli S., Parazzini F., Favilli A. (2023). Maternity Blues: A Narrative Review. J. Pers. Med..

[4] Stewart D.E., Vigod S.N. (2019). Postpartum Depression: Pathophysiology, Treatment, and Emerging Therapeutics. Annu. Rev. Med..

[5] Radoš S.N., Akik B.K., Žutić M., Rodriguez-Muñoz M.F., Uriko K., Motrico E., Moreno-Peral P., Apter G., den Berg M.L. (2024). Diagnosis of peripartum depression disorder: A state-of-the-art approach from the COST Action Riseup-PPD. Compr. Psychiatry.

[6] Dominiak M., Antosik-Wojcinska A.Z., Baron M., Mierzejewski P., Swiecicki L. (2021). Recommendations for the prevention and treatment of postpartum depression. Ginekol. Pol..

[7] World Health Organization (2015). Thinking Healthy: A Manual for Psychosocial Management of Perinatal Depression.

[8] Lindahl V., Pearson J.L., Colpe L. (2005). Prevalence of suicidality during pregnancy and the postpartum. Arch. Womens Ment. Health.

[9] Yahya N.F.S., Teng N.I.M.F., Das S., Juliana N. (2021). Nutrition and physical activity interventions to ameliorate postpartum depression: A scoping review. Asia Pac. J. Clin. Nutr..

[10] Halligan S.L., Murray L., Martins C., Cooper P.J. (2007). Maternal depression and psychiatric outcomes in adolescent offspring: A 13-year longitudinal study. J. Affect. Disord..

[11] Righetti-Veltema M., Bousquet A., Manzano J. (2003). Impact of postpartum depressive symptoms on mother and her 18-month-old infant. Eur. Child Adolesc. Psychiatry.

[12] Righetti-Veltema M., Conne-Perréard E., Bousquet A., Manzano J. (2002). Postpartum depression and mother–infant relationship at 3 months old. J. Affect. Disord..

[13] Moore Simas T.A., Whelan A., Byatt N. (2024). Screening Recommendations and Treatments for Postpartum Depression. JAMA.

[14] Faravelli C., Alessandra Scarpato M., Castellini G., Lo Sauro C. (2013). Gender differences in depression and anxiety: The role of age. Psychiatry Res..

[15] Sadat Z., Abedzadeh-Kalahroudi M., Atrian M.K., Karimian Z., Sooki Z. (2014). The impact of postpartum depression on quality of life in women after child’s birth. Iran. Red. Crescent Med. J..

[16] Gress-Smith J.L., Luecken L.J., Lemery-Chalfant K., Howe R. (2012). Postpartum depression prevalence and impact on infant health, weight, and sleep in low-income and ethnic minority women and infants. Matern. Child. Health J..

[17] O’Brien A.P., McNeil K.A., Fletcher R., Conrad A., Wilson A.J., Jones D., Chan S.W. (2016). New fathers’ perinatal depression and anxiety—Treatment options: An integrative review. Am. J. Mens. Health.

[18] Beck C.T. (2001). Predictors of postpartum depression: An update. Nurs. Res..

[19] Gavin I.N., Gaynes N.B., Lohr N.K., Meltzer-Brody N.S., Gartlehner N.G., Swinson N.T. (2005). Perinatal depression a systematic review of prevalence and incidence. Obstet. Gynecol..

[20] Wang Z., Liu J., Shuai H., Cai Z., Fu X., Liu Y., Xiao X., Zhang W., Krabbendam E., Liu S. (2021). Mapping global prevalence of depression among postpartum women. Transl. Psychiatry.

[21] Li Y., Zhao Q., Cross W.M., Chen J., Qin C., Sun M. (2020). Assessing the quality of mobile applications targeting postpartum depression in China. Int. J. Ment. Health Nurs..

[22] Sohr-Preston S.L., Scaramella L.V. (2006). Implications of timing of maternal depressive symptoms for early cognitive and language development. Clin. Child Fam. Psychol. Rev..

[23] Sainuddin S.S., Norhayati M.N., Abdul Kadir A., Zakaria R. (2023). A 10-year systematic review and meta-analysis of determinants of postpartum depression in the Association of Southeast Asian Nations countries. Med. J. Malays..

[24] Liu X., Wang S., Wang G. (2022). Prevalence and Risk Factors of Postpartum Depression in Women: A Systematic Review and Meta-analysis. J. Clin. Nurs..

[25] Gelaye B., Rondon M.B., Araya R., Williams M.A. (2016). Epidemiology of maternal depression, risk factors, and child outcomes in low-income and middle-income countries. The lancet. Psychiatry.

[26] Parsons C.E., Young K.S., Rochat T.J., Kringelbach M.L., Stein A. (2012). Postnatal depression and its effects on child development: A review of evidence from low- and middle-income countries. Br. Med. Bull..

[27] Fisher J., de Mello M.C., Patel V., Rahman A., Tran T., Holton S., Holmes W. (2012). Prevalence and determinants of common perinatal mental disorders in women in low- and lower-middle-income countries: A systematic review/Prevalence et determinants des troubles mentaux perinataux communs chez les femmes des pays a revenu faible et moyen: Une etude systematique/Prevalencia y determinantes de los trastornos mentales perinatales frecuentes en mujeres en paises de ingresos bajos y medios-bajos: Examen sistematico. Bull. WHO.

[28] Chechko N., Losse E., Frodl T., Nehls S. (2023). Baby blues, premenstrual syndrome and postpartum affective disorders: Intersection of risk factors and reciprocal influences. BJPsych Open.

[29] Chen J., Cross W.M., Plummer V., Lam L., Tang S. (2019). A systematic review of prevalence and risk factors of postpartum depression in Chinese immigrant women. Women Birth J. Aust. Coll. Midwives.

[30] Abdollahi F., Lye M.-S., Zain A.M., Ghazali S.S., Zarghami M. (2011). Postnatal Depression and Its Associated Factors in Women From Different Cultures. Iran. J. Psychiatry Behav. Sci..

[31] Ishikawa N., Goto S., Murase S., Kanai A., Masuda T., Aleksic B., Usui H., Ozaki N. (2011). Prospective study of maternal depressive symptomatology among Japanese women. J. Psychosom. Res..

[32] Suzuki S. (2024). Prevention of Postpartum Depression by Multidisciplinary Collaboration in Japan. JMA J..

[33] Shafiei T., Small R., McLachlan H. (2015). Immigrant Afghan women’s emotional well-being after birth and use of health services in Melbourne, Australia. Midwifery.

[34] Canada Statistics Immigrant Population in Canada, 2016 Census of Population. https://www150.statcan.gc.ca/n1/pub/11-627-m/11-627-m2017028-eng.htm.

[35] Zhao X.H., Zhang Z.H. (2020). Risk factors for postpartum depression: An evidence-based systematic review of systematic reviews and meta-analyses. Asian J. Psychiatry.

[36] Agrawal I., Mehendale A.M., Malhotra R. (2022). Risk Factors of Postpartum Depression. Cureus.

[37] Guintivano J., Sullivan P.F., Stuebe A.M., Penders T., Thorp J., Rubinow D.R., Meltzer-Brody S. (2018). Adverse life events; psychiatric history, and biological predictors of postpartum depression in an ethnically diverse sample of postpartum women. Psychol. Med..

[38] Maguire J., Mody I. (2016). Behavioral Deficits in Juveniles Mediated by Maternal Stress Hormones in Mice. Neural Plast..

[39] Murgatroyd C.A., Taliefar M., Bradburn S., Carini L.M., Babb J.A., Nephew B.C. (2015). Social stress during lactation, depressed maternal care, and neuropeptidergic gene expression. Behav. Pharmacol..

[40] Baird H., Harris R.A., Santos H.P. (2023). The Effects of Maternal Perinatal Depression on Child IQ: A Systematic Review. Matern. Child Health J..

[41] Horsch A., Garthus-Niegel S., Ayers S., Chandra P., Hartmann K., Vaisbuch E., Lalor J. (2024). Childbirth-related posttraumatic stress disorder: Definition, risk factors, pathophysiology, diagnosis, prevention, and treatment. Am. J. Obstet. Gynecol..

[42] Forty L., Jones L., Macgregor S., Caesar S., Cooper C., Hough A., Dean L., Dave S., Farmer A., McGuffin P. (2006). Familiality of Postpartum Depression in Unipolar Disorder: Results of a Family Study. Am. J. Psychiatry.

[43] Murphy-Eberenz K., Zandi P.P., March D., Crowe R.R., Scheftner W.A., Alexander M., McInnis M.G., Adams P., DePaulo J.R., Miller E.B. (2006). Is perinatal depression familial?. J. Affect. Disord..

[44] Payne J.L., Maguire J. (2019). Pathophysiological mechanisms implicated in postpartum depression. Front. Neuroendocr..

[45] Mehta D., Newport D.J., Frishman G., Kraus L., Rex-Haffner M., Ritchie J.C., Lori A., Knight B.T., Stagnaro E., Ruepp A. (2014). Early predictive biomarkers for postpartum depression point to a role for estrogen receptor signaling. Psychol. Med..

[46] Binder E.B., Newport D.J., Zach E.B., Smith A.K., Deveau T.C., Altshuler L.L., Cohen L.S., Stowe Z.N., Cubells J.F. (2010). A serotonin transporter gene polymorphism predicts peripartum depressive symptoms in an at-risk psychiatric cohort. J. Psychiatr. Res..

[47] Pinheiro R.T., Coelho F.M., Silva R.A., Pinheiro K.A., Oses J.P., Quevedo Lde Á., Souza L.D., Jansen K., Zimmermann Peruzatto J.M., Manfro G.G. (2013). Association of a serotonin transporter gene polymorphism (5-HTTLPR) and stressful life events with postpartum depressive symptoms: A population-based study. J. Psychosom. Obs. Gynaecol..

[48] Yang Y., Fang M., Du X., Hu Z. (2017). Lucky gene 5-HTTLPR and postpartum depression: A systematic review. Neuro Endocrinol. Lett..

[49] Landoni M., Missaglia S., Tavian D., Ionio C., Di Blasio P. (2022). Influence of 5-HTTLPR polymorphism on postpartum depressive and posttraumatic symptoms. Psychiatr. Genet..

[50] Doornbos B., Dijck-Brouwer D.A.J., Kema I.P., Tanke M.A.C., van Goor S.A., Muskiet F.A.J., Korf J. (2009). The development of peripartum depressive symptoms is associated with gene polymorphisms of MAOA, 5-HTT and COMT. Prog. Neuro-Psychopharmacol. Biol. Psychiatry.

[51] Sacher J., Rekkas P.V., Wilson A.A., Houle S., Romano L., Hamidi J., Rusjan P., Fan I., Stewart D.E., Meyer J.H. (2015). Relationship of monoamine oxidase-A distribution volume to postpartum depression and postpartum crying. Neuropsychopharmacology.

[52] Ma J., Huang Z., Wang S., Zheng S., Duan K. (2019). Postpartum depression: Association with genetic polymorphisms of noradrenaline metabolic enzymes and the risk factors. Nan Fang Yi Ke Da Xue Xue Bao.

[53] Pariante C.M., Lightman S.L. (2008). The HPA axis in major depression: Classical theories and new developments. Trends Neurosci..

[54] Chai Y., Li Q., Wang Y., Tao E., Asakawa T. (2022). The Value of HPA Axis Hormones as Biomarkers for Screening and Early Diagnosis of Postpartum Depression: Updated Information About Methodology. Front. Endocrinol..

[55] Dennis C.L., Ross L.E., Herxheimer A. (2008). Oestrogens and progestins for preventing and treating postpartum depression. Cochrane Database Syst. Rev..

[56] Kettunen P., Koistinen E., Hintikka J., Perheentupa A. (2022). Oestrogen therapy for postpartum depression: Efficacy and adverse effects. A double-blind, randomized, placebo-controlled pilot study. Nord. J. Psychiatry.

[57] Sundström-Poromaa I., Comasco E., Sumner R., Luders E. (2020). Progesterone-Friend or foe?. Front. Neuroendocrinol..

[58] Henshaw E.J. (2023). Breastfeeding and Postpartum Depression: A Review of Relationships and Potential Mechanisms. Curr. Psychiatry Rep..

[59] Bell A.F., Erickson E.N., Carter C.S. (2014). Beyond labor: The role of natural and synthetic oxytocin in the transition to motherhood. J. Midwifery Women’s Health.

[60] Thul T.A., Corwin E.J., Carlson N.S., Brennan P.A., Young L.J. (2020). Oxytocin and postpartum depression: A systematic review. Psychoneuroendocrinology.

[61] Pedersen C.A., Johnson J.L., Silva S., Bunevicius R., Meltzer-Brody S., Hamer R.M., Leserman J. (2007). Antenatal thyroid correlates of postpartum depression. Psychoneuroendocrinology.

[62] Bloch M., Schmidt P.J., Danaceau M., Murphy J., Nieman L., Rubinow D.R. (2000). Effects of Gonadal Steroids in Women with a History of Postpartum Depression. Am. J. Psychiatry.

[63] Bloch M., Daly R.C., Rubinow D.R. (2003). Endocrine factors in the etiology of postpartum depression. Compr. Psychiatry.

[64] Schiller C.E., Meltzer-Brody S., Rubinow D.R. (2015). The role of reproductive hormones in postpartum depression. CNS Spectr..

[65] Stuebe A.M., Grewen K., Meltzer-Brody S. (2013). Association Between Maternal Mood and Oxytocin Response to Breastfeeding. J. Women’s Health.

[66] Mah B.L., Van Ijzendoorn M.H., Out D., Smith R., Bakermans-Kranenburg M.J. (2017). The Effects of Intranasal Oxytocin Administration on Sensitive Caregiving in Mothers with Postnatal Depression. Child Psychiatry Hum. Dev..

[67] Whitley J., Wouk K., Bauer A.E., Grewen K., Gottfredson N.C., Meltzer-Brody S., Propper C., Mills-Koonce R., Pearson B., Stuebe A. (2020). Oxytocin during breastfeeding and maternal mood symptoms. Psychoneuroendocrinology.

[68] Cheng B., Hu X., Roberts N., Zhao Y., Xu X., Zhou Y., Tan X., Chen S., Meng Y., Wang S. (2022). Prolactin mediates the relationship between regional gray matter volume and postpartum depression symptoms. J. Affect. Disord..

[69] Wiciński M., Malinowski B., Puk O., Socha M., Słupski M. (2020). Methyldopa as an inductor of postpartum depression and maternal blues: A review. Biomed. Pharmacother. Biomed. Pharmacother..

[70] Ikeda M., Kamibeppu K. (2013). Measuring the risk factors for postpartum depression: Development of the Japanese version of the Postpartum Depression Predictors Inventory-Revised (PDPI-R-J). BMC Pregnancy Childbirth.

[71] Beck C.T., Gable R.K. (2000). Postpartum depression screening scale: Development and psychometric testing. Nurs. Res..

[72] Mirsalimi F., Ghofranipour F., Noroozi A., Montazeri A. (2020). The postpartum depression literacy scale (PoDLiS): Development and psychometric properties. BMC Pregnancy Childbirth.

[73] Alves S., Fonseca A., Canavarro M.C., Pereira M. (2018). Preliminary Psychometric Testing of the Postpartum Depression Predictors Inventory-Revised (PDPI-R) in Portuguese Women. Matern. Child Health J..

[74] Records K., Rice M., Beck C.T. (2007). Psychometric assessment of the postpartum depression predictors inventory-revised. J. Nurs. Meas..

[75] Bagheri P., Rostami M. (2021). Postpartum depression and suicide in Iran. Womens Health.

[76] Lee Y.L., Tien Y., Bai Y.S., Lin C.K., Yin C.S., Chung C.H., Sun C.A., Huang S.H., Huang Y.C., Chien W.C. (2022). Association of Postpartum Depression with Maternal Suicide: A Nationwide Population-Based Study. Int. J. Environ. Res. Public. Health.

[77] Huang R.S., Spence A.R., Abenhaim H.A. (2024). Non-Obstetric Maternal Mortality Trends by Race in the United States. Matern. Child Health J..

[78] Moya E., Larson L.M., Stewart R.C., Fisher J., Mwangi M.N., Phiri K.S. (2022). Reliability and validity of depression anxiety stress scale (DASS)-21 in screening for common mental disorders among postpartum women in Malawi. BMC Psychiatry.

[79] Braverman J., Roux J. (1978). Screening for the patient at risk for postpartum depression. Obs. Gynecol..

[80] Beck C.T. (2002). Revision of the postpartum depression predictors inventory. J. Obs. Gynecol. Neonatal Nurs..

[81] Beck C.T. (1996). A meta-analysis of predictors of postpartum depression. Nurs. Res..

[82] Beck A.T., Steer R.A. (1990). Manual for the Beck Anxiety Inventory.

[83] Conradt E., Manian N., Bornstein M.H. (2012). Screening for Depression in the Postpartum using the Beck Depression Inventory-II: What Logistic Regression Reveals. J. Reprod. Infant. Psychol..

[84] Su K.P., Chiu T.H., Huang C.L., Ho M., Lee C.C., Wu P.L., Lin C.Y., Liau C.H., Liao C.C., Chiu W.C. (2007). Different cutoff points for different trimesters? The use of Edinburgh Postnatal Depression Scale and Beck Depression Inventory to screen for depression in pregnant Taiwanese women. Gen. Hosp. Psychiatry.

[85] Cox J.L., Holden J.M., Sagovsky R. (1987). Detection of postnatal depression. Development of the 10-item Edinburgh Postnatal Depression Scale. Br. J. Psychiatry.

[86] Oliveira T.A., Luzetti G.G., Rosalém M.M., Neto C.M. (2022). Screening of Perinatal Depression Using the Edinburgh Postpartum Depression Scale. Rev. Bras. De Ginecol. E Obs./RBGO Gynecol. Obstet..

[87] Park S.H., Kim J.I. (2023). Predictive validity of the Edinburgh postnatal depression scale and other tools for screening depression in pregnant and postpartum women: A systematic review and meta-analysis. Arch. Gynecol. Obstet..

[88] Levis B., Negeri Z., Sun Y., Benedetti A., Thombs B.D. (2020). Accuracy of the Edinburgh Postnatal Depression Scale (EPDS) for screening to detect major depression among pregnant and postpartum women: Systematic review and meta-analysis of individual participant data. BMJ.

[89] Adouard F., Glangeaud-Freudenthal N.M.C., Golse B. (2005). Validation of the Edinburgh postnatal depression scale (EPDS) in a sample of women with high-risk pregnancies in France. Arch. Womens Ment. Health.

[90] Kubota C., Okada T., Aleksic B., Nakamura Y., Kunimoto S., Morikawa M., Shiino T., Tamaji A., Ohoka H., Banno N. (2014). Factor structure of the Japanese version of the Edinburgh postnatal depression scale in the postpartum period. PLoS ONE.

[91] Small R., Lumley J., Yelland J., Brown S. (2007). The performance of the Edinburgh postnatal depression scale in english speaking and non-English speaking populations in Australia. Soc. Psychiatry Psychiatr. Epidemiol..

[92] Kotz J., Marriott R., Reid C. (2021). The EPDS and Australian Indigenous women: A systematic review of the literature. Women Birth J. Aust. Coll. Midwives.

[93] Chávez-Tostado M., Chávez-Tostado K.V., Cervantes-Guevara G., Cervantes-Cardona G., Hernandez-Corona D.M., González-Heredia T., Méndez-del Villar M., Corona-Meraz F.I., Guzmán-Ornelas M.O., Barbosa-Camacho F.J. (2023). Breastfeeding Practices and Postpartum Depression in Mexican Women during the COVID-19 Pandemic: A Cross-Sectional Study. Medicina.

[94] King P.A.L. (2012). Replicability of structural models of the Edinburgh Postnatal Depression Scale (EPDS) in a community sample of postpartum African American women with low socioeconomic status. Arch. Womens Ment. Health.

[95] Lovibond P.F., Lovibond S.H. (1995). The structure of negative emotional states: Comparison of the Depression Anxiety Stress Scales (DASS) with the Beck Depression and Anxiety Inventories. Behav. Res. Ther..

[96] Price DA M., Middleton M.M., Matthey AA P.S., Goldfeld P.S., Kemp P.L., Orsini M.F. (2021). A comparison of two measures to screen for mental health symptoms in pregnancy and early postpartum: The Matthey Generic Mood Questionnaire and the Depression, Anxiety, Stress Scales short-form. J. Affect. Disord..

[97] Maxwell D., Robinson S.R., Rogers K. (2018). “I keep it to myself”: A qualitative meta-interpretive synthesis of experiences of postpartum depression among marginalised women. Health Soc. Care Community.

[98] Luoma I., Tamminen T., Kaukonen P., Laippala P., Puura K., Salmelin R., Almqvist F. (2001). Longitudinal study of maternal depressive symptoms and child well-being. J. Am. Acad. Child. Adolesc. Psychiatry.

[99] Schechter J.C., Brennan P.A., Smith A.K., Stowe Z.N., Newport D.J., Johnson K.C. (2016). Maternal prenatal psychological distress and preschool cognitive functioning: The protective role of positive parental engagement. J. Abnorm. Child. Psychol..

[100] Evans J., Melotti R., Heron J., Ramchandani P., Wiles N., Murray L., Stein A. (2011). The timing of maternal depressive symptoms and child cognitive development: A longitudinal study. J. Child. Psychol. Psychiatry.

[101] Faleschini S., Rifas-Shiman S., Tiemeier H., Oken E., Hivert M.F. (2019). Associations of prenatal and postnatal maternal depressive symptoms with ofspring cognition and behavior in Mid-Childhood: A prospective cohort study. Int. J. Environ. Res. Public Health.

[102] Walker L.O. (2024). Maternal postpartum health and its impact on health and development of young children. Womens Health Nurs..

[103] Murray L., Arteche A., Fearon P., Halligan S., Croudace T., Cooper P. (2010). The efects of maternal postnatal depression and child sex on academic performance at age 16 years: A developmental approach. J. Child. Psychol. Psychiatry.

[104] Amani B., Merza D., Savoy C., Streiner D., Bieling P., Ferro M.A., Van Lieshout R.J. (2021). Peer-Delivered Cognitive-Behavioral Therapy for Postpartum Depression: A Randomized Controlled Trial. J. Clin. Psychiatry.

[105] Li X., Laplante D.P., Paquin V., Lafortune S., Elgbeili G., King S. (2022). Effectiveness of cognitive behavioral therapy for perinatal maternal depression, anxiety and stress: A systematic review and meta-analysis of randomized controlled trials. Clin. Psychol. Rev..

[106] Spelke M.B., Paul R., Blette B.S., Meltzer-Brody S., Schiller C.E., Ncheka J.M., Kasaro M.P., Price J.T., Stringer J.S.A., Stringer E.M. (2022). Interpersonal therapy versus antidepressant medication for treatment of postpartum depression and anxiety among women with HIV in Zambia: A randomized feasibility trial. J. Int. AIDS Soc..

[107] Guille C., Newman R., Fryml L.D., Lifton C.K., Epperson C.N. (2013). Management of postpartum depression. J. Midwifery Womens Health.

[108] (2023). Treatment and Management of Mental Health Conditions During Pregnancy and Postpartum: ACOG Clinical Practice Guideline No. 5. Obstet. Gynecol..

[109] Kroska E.B., Stowe Z.N. (2020). Postpartum Depression: Identification and Treatment in the Clinic Setting. Obs. Gynecol. Clin. N. Am..

[110] Pettman D., O’Mahen H., Blomberg O., Svanberg A.S., von Essen L., Woodford J. (2023). Effectiveness of cognitive behavioural therapy-based interventions for maternal perinatal depression: A systematic review and meta-analysis. BMC Psychiatry.

[111] Dennis C.L., Ravitz P., Grigoriadis S., Jovellanos M., Hodnett E., Ross L., Zupancic J. (2012). The effect of telephone-based interpersonal psychotherapy for the treatment of postpartum depression: Study protocol for a randomized controlled trial. Trials.

[112] Prevatt B.S., Lowder E.M., Desmarais S.L. (2018). Peer-support intervention for postpartum depression: Participant satisfaction and program effectiveness. Midwifery.

[113] Dennis C.L. (2010). Postpartum depression peer support: Maternal perceptions from a randomized controlled trial. Int. J. Nurs. Stud..

[114] Cornett E.M., Rando L., Labbé A.M., Perkins W., Kaye A.M., Kaye A.D., Viswanath O., Urits I. (2021). Brexanolone to Treat Postpartum Depression in Adult Women. Psychopharmacol. Bull..

[115] Deligiannidis K.M., Meltzer-Brody S., Maximos B., Peeper E.Q., Freeman M., Lasser R., Bullock A., Kotecha M., Li S., Forrestal F. (2023). Zuranolone for the Treatment of Postpartum Depression. Am. J. Psychiatry.

[116] Hutcherson T.C., Cieri-Hutcherson N.E., Gosciak M.F. (2020). Brexanolone for postpartum depression. Am. J. Health Syst. Pharm..

[117] Suryawanshi O., Pajai S. (2022). A Comprehensive Review on Postpartum Depression. Cureus.

