# Supplementary material for: Postpartum Depression Epidemiology, Risk Factors, Diagnosis, and Management: An Appraisal of the Current Knowledge and Future Perspectives
Source: J Clin Med. 2025 Apr 1;14(7):2418. doi: 10.3390/jcm14072418 (PMC11989329; doi:10.3390/jcm14072418)
Supplement: Supplementary file 1 [file jcm-14-02418-s001.zip › jcm-3536330-supplementary.pdf]

Supplementary Table S1. Search threads.

Search thread 1	(“postpartum depression” OR (“postpartum” AND “depression”)) OR “maternal mental health” OR “maternal behavior” OR (“well*being” AND postpartum) AND (“epidemiology” OR “incidence”) AND (“risk factors” OR “risks” OR “pathophysiology”) AND (“diagnosis” OR “symptoms” OR “instrument”) AND (“child” OR “newborn” OR “kid”) AND (“management” OR “intervention”)
Search thread 2	(“postpartum depression” OR (“perinatal” AND “depression”) OR “maternal mental health” OR “maternal” AND “mental health” OR (“well*being” AND postpartum)) AND (“epidemiology” OR “prevalence”) AND (“risk factors” OR “pathology”) AND (“diagnosis” OR “symptoms” OR “scoring”) AND (“child” OR “newborn” OR “kid”) AND (“management” OR “intervention”)
Search thread 3	(“postpartum depression” OR (“perinatal” AND “depression”) OR “maternal mental health” OR “maternal” AND “mental health” OR (“well*being” AND postpartum)) AND (“epidemiology” OR “prevalence”) AND (“risk factors” OR “pathology”) AND (“diagnosis” OR “symptoms” OR “scoring”) AND (“management” OR “intervention” OR “ECT” OR “medication” OR “cognitive-behavioral therapy” OR “interpersonal therapy”)
